# Utilization of Landsat-8 data for the estimation of carrot and maize crop water footprint under the arid climate of Saudi Arabia

**DOI:** 10.1371/journal.pone.0192830

**Published:** 2018-02-12

**Authors:** Rangaswamy Madugundu, Khalid A. Al-Gaadi, ElKamil Tola, Abdalhaleem A. Hassaballa, Ahmed G. Kayad

**Affiliations:** 1 Precision Agriculture Research, King Saud University, Riyadh, Saudi Arabia; 2 Department of Agricultural Engineering, College of Food and Agriculture Sciences, King Saud University, Riyadh, Saudi Arabia; Washington State Univeristy, UNITED STATES

## Abstract

The crop Water Footprint (WF) can provide a comprehensive knowledge of the use of water through the demarcation of the amount of the water consumed by different crops. The WF has three components: green (WF_g_), blue (WF_b_) and grey (WF_gr_) water footprints. The WF_g_ refers to the rainwater stored in the root zone soil layer and is mainly utilized for agricultural, horticultural and forestry production. The WF_b_, however, is the consumptive use of water from surface or groundwater resources and mainly deals with irrigated agriculture, industry, domestic water use, etc. While the WF_gr_ is the amount of fresh water required to assimilate pollutants resulting from the use of fertilizers/agrochemicals. This study was conducted on six agricultural fields in the Eastern region of Saudi Arabia, during the period from December 2015 to December 2016, to investigate the spatiotemporal variation of the WF of silage maize and carrot crops. The WF of each crop was estimated in two ways, namely agro-meteorological (WF_Agro_) and remote sensing (WF_RS_) methods. The blue, green and grey components of WF_Agro_ were computed with the use of weather station/Eddy covariance measurements and field recorded crop yield datasets. The WF_RS_ estimated by applying surface energy balance principles on Landsat-8 imageries. However, due to non-availability of Landsat-8 data on the event of rainy days, this study was limited to blue component (WF_RS-b_). The WF_Agro_ of silage maize was found to range from 3545 m^3^ t^-1^ to 4960 m^3^ t^-1^; on an average, the WF_Agro-g_, WF_Agro-b_, and WF_Agro-gr_ are composed of < 1%, 77%, and 22%, respectively. In the case of carrot, the WF_Agro_ ranged between 297 m^3^ t^-1^ and 502 m^3^ t^-1^. The WF_Agro-g_ of carrot crop was estimated at <1%, while WF_Agro-b_ and WF_Agro-gr_ was 67% and 32%, respectively. The WF_Agro-b_ is occupied as a major portion in WF of silage maize (77%) and carrot (68%) crops. This is due to the high crop water demand combined with a very erratic rainfall, the irrigation is totally provided using groundwater delivered by center pivot irrigation systems. On the other hand, the WF_RS-b_ estimated using Landsat-8 data was varied from 276 (±73) m^3^ t^-1^ (carrot) and 2885 (±441) m^3^ t^-1^ (silage maize). The variation (RMSE) between WF_RS-b_ and WF_Agro-b_ was about 17% and 14% for silage maize and carrot crops, respectively.

## Introduction

Although a significant amount of water is being consumed in the industrial and domestic sectors, the agricultural sector is considered as the largest consumer of water with 80% of water consumption worldwide [1]. Therefore, unless the current water management practices become dramatically wiser; many parts of the world will face rigorous competition for water among agriculture, energy, industry and civil activities [2].

Water scarcity, which refers to the lack of satisfactory available water resources to meet the water needs within a particular region, can be classified into two types, physical and economic [3]. The physical water scarcity, as per Srinivasan et al. [3], results from natural water resources that are inadequate to meet the demand of a certain area; however, the economic water scarcity results from poor handling of accessible water resources. Water scarcity is considered as one of the most critical problems facing many societies worldwide, and is directly interlinked to the food sector as over 80% of the world water withdrawal is to meet the requirements of the increasing population and the continuing development [4]. Due to water scarcity, many countries are forced to import food from abroad to meet the demand of its own people.

Water Footprint (WF), originated from the conception of Virtual Water (VW) [5], can be defined as being the total volume of freshwater used to produce the goods and services consumed by the individual or community or produced by the business, and is measured as volume of water consumed (evaporated) and/or polluted per unit of time [6,7]. Therefore, WF is an effective means that is used to quantify stress on water resources to address global, regional, national and local water scarcities. In this text, WF of a crop is defined as being the volume of freshwater used to produce a unit of a specific agricultural product (m^3^ t^-1^) throughout the period of its production [7,8]. Crop WF provides an effective tool to investigate the linkage between food and water resources as a function of climate, soil and agricultural practices [9]. In general, WF analysis connects a wide range of sectors and issues and provides a multi-disciplinary scope for efficient management of water resources [10].

The three components of the WF that provide a thorough insight of the use of water through the demarcation of the consumed water are the green, the blue, and the grey WF. The blue WF (WF_b_) refers to the consumption of blue water resources (surface and groundwater) by a product throughout its water demand stages. Water consumption is defined as being the loss of water from the available ground and/or surface water in a catchment area. Losses occur when water evaporates, returns to another catchment area (i.e. the sea) or is incorporated into a product [7]. The green WF (WF_g_) is the consumption of green water resources (rainwater as far as it is not considered as run-off). The grey WF (WF_gr_), however, refers to the pollution and is defined as being the volume of freshwater required to absorb the load of pollutant concentrations in a given natural environment.

The blue water resources are generally scarcer and costlier compared to the green water resources, which are valid reasons to place most of the emphasis and focus on only the blue water footprint. However, the green water resources are also limited and, therefore, are considered scarce, which provides an argument to account for the green WF as well. In addition, the green water can be substituted by the blue water and, in agriculture, the reverse can also take a place indicating that a complete knowledge can be obtained by accounting for both types of water.

After the International Expert Meeting on virtual water (VW) Trade held in December 2002 and the special session on VW Trade and Geopolitics during the Third World Water Forum held in March 2003, many studies have highlighted WF as a tool for global, regional and national water savings [8, 11–16], others have calculated the WF on a global scale [6,17]. Many studies have focused on WF and VW trade among countries with primary crops at sub-national/local levels in conjunction with variations in climate, soil and other factors [18]. Studies have been accomplished, at the national level, on the use of water for crop production, municipal water consumption and the flow of water in countries, such as China [19,20], India [18], the Netherlands [21], the UK [22], Indonesia [23] and Nepal [24]. However, such studies are very limited in Saudi Arabia [25–27], and are not available even at the level of fields or farms, which are experiencing dynamic climates, scarce water reserves, and limited rainfall.

On the other hand, existing methods calculate the WF using data from national statistics, reports and climatic databases [7,19,24,25]. Remote sensing techniques provide the possibility of mapping the WF of a specific crop through the use of models, such as crop growth, productivity, and crop water use models. However, the use of remote sensing data in the quantification of WF is limited [28–30]. Therefore, this study was designed to bridge the gap in knowledge existed in the area of WF in the Kingdom of Saudi Arabia by quantifying and analyzing the spatial variation of WF of maize and carrot crops, cultivated during the period from December 2015 to December 2016, in the Eastern region of the Kingdom. The WF of silage maize and carrot crops was estimated in two ways, namely: agro-meteorological (WF_Agro_) and remote sensing (WF_RS_) methods. The green (WF_Agro-g_), blue (WF_Agro-b_) and grey (WF_Agro-gr_) components of WF_Agro_ were computed with the use of weather station/Eddy covariance measurements and field recorded crop yield datasets. The WF_RS_ estimated by applying surface energy balance principles on Landsat-8 imageries.

Landsat images are widely used in ET mapping and in the estimation of the WF_g_ and the WF_b_. However, the sparse and erratic rainfall conditions over the study site, resulted in a lack of Landsat-8 images coinciding with the rainy days. Hence, this study was limited to the blue component (WF_RS-b_) of the WF_RS_ approach. Moreover, the WF_Agro_ estimates were used as a reference for the accuracy assessment of Landsat-8 derived WF_RS-b_ in conjunction with the climatic conditions and cropping patterns.

## Materials and methods

### Study area

**Fig 1:**
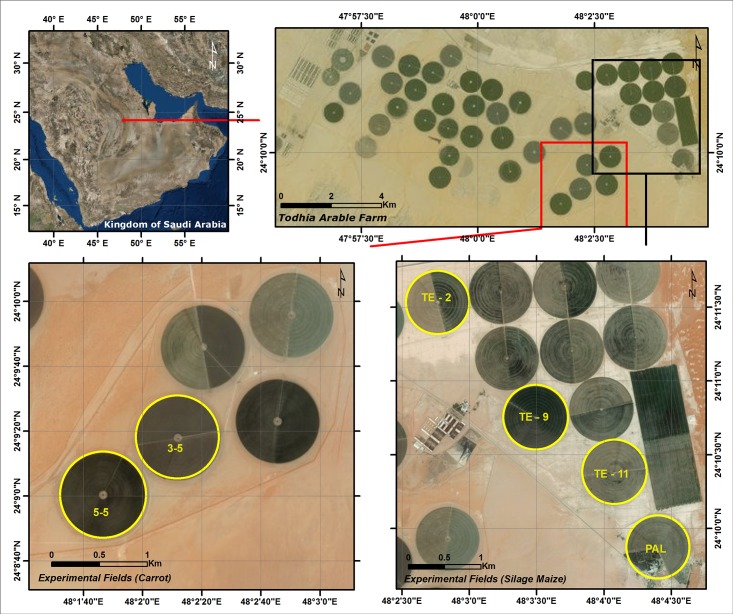
Location map of the study area (image source: ArcGIS base map from http://services.arcgisonline.com/arcgis/services).

The study was carried out in six 50 ha agricultural fields that were part of the 47 fields of the Todhia Arable Farm (TAF) located about 250 km Southeast of Riyadh, the capital city of Saudi Arabia, at coordinates of 24° 11′ 00″ E and 48° 56′ 14.6″ N (Fig 1). TAF was in an arid climate with hot summers (40 ± 2 °C) and cold to moderate winters (15 ± 3 °C), with a mean air temperature of 35 °C. The annual rainfall was about 90 mm, most of which occurred in the period from November to February. Due to the high crop water demand combined with highly erratic rainfall, irrigation is totally provided using groundwater delivered by center pivot irrigation systems. The major crops cultivated in the study area were forages (alfalfa, Rhodes grass and corn) and vegetable crops (carrot and lettuce). Depending on the demand, vegetable crops were cultivated throughout the year.

### Field data

For the determination of WF of carrot and maize crops, a field survey was conducted to understand the cropping pattern of the experimental farm and to develop the sampling strategy (permission to conduct the study was issued by the Farm Manager, Mr. Alan King). Carrot crop was cultivated throughout the year; while, maize crop was cultivated twice a year (March–June, and July–November). Out of the 47 fields, maize crop was cultivated in 23 fields and carrot was cultivated in only seven fields during the period from December 2015 to December 2016. For this study, six center pivot irrigated fields were considered as sample fields, four fields (TE-2, TE-9, TE-11, PAL) were designated for silage maize and the remaining two for carrot (3–5, 5–5). Datasets pertaining agricultural practices, such as sowing and harvesting dates, crop growth stages, amount of applied irrigation water, agro-climatic data and crop yields were obtained for the whole crop growth period. Except for the agro-climatic data which was extracted from an Eddy Covariance system installed in the farm, the data sets were taken from the records of the experimental farm.

### Water footprint of agricultural crops

**Fig 2:**
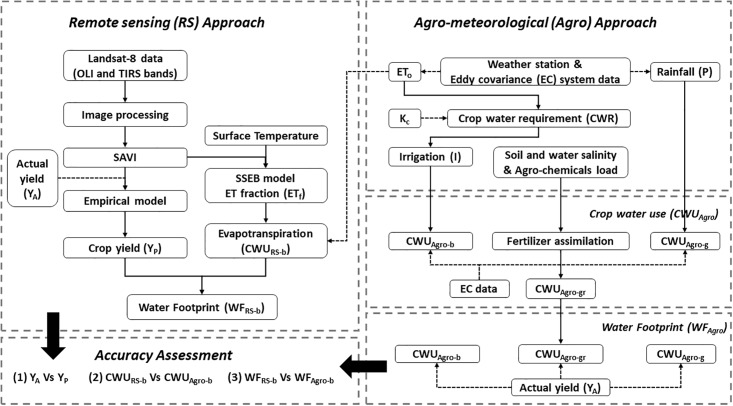
Methodological flowchart—Water Footprint of crops from remote sensing data and empirical approach.

The methodological flow of determining the WF of agricultural crops is provided in Fig 2. The agro-meteorology (WF_Agro_) and remote sensing (WF_RS_) methods were applied to estimate the WF of silage maize and carrot crops. The WF_Agro_ is computed with the use of empirical equations. Weather station/Eddy covariance system measured parameters (temperature, precipitation, evapotranspiration, etc.,) and the field measured crop yield datasets were used as input parameters. The WF_Argo-g_ and WF_Argo-b_ were considered as the amount of water consumed by the crop, while the WF_Argo-gr_ is the amount of water needed for leaching requirement and to assimilate possible applied agro-chemicals [16].

### Agro-meteorological (empirical) approach

#### Crop water requirement

Crop water requirement (CWR) was calculated by multiplying the reference crop evapotranspiration (ET_o_) by the crop coefficient (K_c_), as in Eq (1) described by Savva and Frenken [31]. In this study, the CWR was assumed to be equal to the actual crop evapotranspiration (ET_c_) and there were no water limitations to crop growth so that the CWR was fully met. However, the crop type, variety and the developmental stage of the crop significantly affect the ET_c_. The K_c_ values listed in FAO [32] were used in this study (Table 1).

$$CWR={ET}_{c}={ET}_{o}\times K_{c}$$ (1)

where, ET_c_ is the crop evapotranspiration (mm d^-1^), K_c_ is the crop coefficient and ET_o_ is the reference crop evapotranspiration (mm d^-1^).

**Table 1:** Season and crop wise length of the growth period (lgp) and K_c_ values (given in parenthesis) taken from [32].

Crop	Season	Field ID	Sowing	Harvesting	Length of the growth period (K_c_ values)				
					Initial	Developmental	Middle	Late	Total
Carrot	Winter	3-5(S)	September/October	December	20 (0.45)	30 (0.80)	40 (1.10)	20 (0.95)	110
		5–5 (S)	November/December	March					
	Summer	3–5 (N)	May	September	30 (0.45)	40 (0.80)	60 (1.10)	20 (0.95)	140
		5–5 (N)	June	October					
Silage Maize	Spring	TE-11	April	July	25 (0.45)	40 (0.80)	40 (1.15)	15 (1.00)	120
		PAL							
	Summer	TE-2	July	October	20 (0.45)	35 (0.80)	40 (1.15)	30 (1.00)	125
		TE-9							

Climatic variations, such as temperature, sunshine, wind speed, and humidity are the controlling factors for *K*_*c*_ and *ET*_*o*_. The ET_o_ (mm d^-1^) was estimated using the formula described in ASCE-EWRI [33] as shown in Eq (2).

$${ET}_{o}=\frac{0.408\Delta (R_{n}-G)+\gamma (\frac{900}{\left(T+273\right)})u_{2}(e_{s}-e_{a})}{\Delta +\gamma (1+0.34u_{2})}$$ (2)

where, R_n_ is the measured net irradiance at the crop canopy (MJ m^-2^ d^-1^), G is the soil heat flux density (MJ m^-2^), T is the mean daily air temperature (°C), U_2_ is the mean daily wind speed at 2 m height (m s^-1^), *e*_*s*_ is the saturated vapor pressure (kPa), *e*_*a*_ is the mean actual vapor pressure (kPa), Δ is the slope of the saturation vapor pressure—temperature-pressure curve (kPa °C^-1^) and γ is the psychometric constant (kPa °C^-1^).

Effective rainfall (*P*_*eff*_) refers to the percentage of rainfall, which becomes available to crops. A number of factors can influence the effective rainfall including the amount and density of the rainfall, soil texture and the bulk density and the topography and the slope of the study area. The *P*_*eff*_ was calculated using Eq (3) following Kuo et al. [34].

$$P_{eff}=P_{tot}\frac{125-0.2P_{tot}}{125}$$ (3)

where, *P*_*eff*_ is the effective rainfall (mm) and *P*_*tot*_ is the total rainfall (mm).

Soil salinity is a common aspect of irrigated agriculture over hyper-arid regions salinity due to evaporation of irrigation water. Therefore, to improve/protect soil quality, leaching out the accumulated salts from the soil profile is essential by applying an excess amount of water at the beginning of the growing season. The amount of water required for leaching, the leaching requirement (LR), is calculated from the Eq (5) as described in FAO [32].

$$LR=\frac{{EC}_{w}}{5\;({EC}_{e})-({EC}_{w})}$$ (4)

where, EC_w_ is salinity of the applied irrigation water (dS m^-1^), EC_e_ is soil salinity measured from the soil saturation extract. In addition to LR, based on the amounts of CWR and P_eff_, the agricultural water requirement for each field was estimated using Eq (5) as described by Chowdhury et al. [35].

$$Q=\sum_{i=1}^{n}A_{i}({ET}_{ci}-P_{eff})\times 10$$ (5)

where, Q is the monthly agricultural water requirement of a field or irrigation scheme (m^3^ d^-1^), *i* is the crop index, A_i_ is the field area (ha), ET_c_ is the crop evapotranspiration (mm d^-1^) and P_eff_ is the effective rainfall (mm d^-1^). Fully automated sprinkler irrigation system was used to achieve high irrigation application efficiency. The efficiency of the sprinkler system was fixed at 70% as described in Al-Zeid et al. [36] for the application of irrigation water to the crops in the experimental fields.

#### Crop water use (CWU)

The green and blue components of the CWU (m^3^ ha^-1^) were calculated from the accumulation of the daily evapotranspiration (ET, mm d^-1^) over the entire growing period. They were calculated using Eqs (6) and (7) according to Chapagain and Hoekstra [37] as follows:

$${CWU}_{green}=10\times \sum_{d=1}^{lgp}{ET}_{green}$$ (6)

$${CWU}_{blue}=10\times \sum_{d=1}^{lgp}{ET}_{blue}$$ (7)

where, *lgp* is the length of the growth period of the studied crop; ET_green_ is the green water evapotranspiration and ET_blue_ is the blue water evapotranspiration; CWU_green_ is the total rainwater evaporated from the field during the growing period and CWU_blue_ is the total irrigation water evaporated from the field.

$$ET_{green}=\text{min}(ET_{c},\;P_{eff})\;\;[length/time]$$ (8)

$$ET_{blue}=\text{max}(0,\;ET_{c}-P_{eff})\;\;[length/time]$$ (9)

where, the ET_green_, i.e. evapotranspiration of rainfall water, is equal to the minimum difference between the total ET_c_ and P_eff_. The ET_blue_, however, is equal to the maximum difference between the total ET_c_ and P_eff_).

The grey assimilation water use (AWU_grey_, m^3^ ha^-1^), the amount of water is used for the leaching (wash out) of salts from the rooting zone. The AWU_grey_ was calculated using Eq (10), by dividing the pollutant load (L_leached_, in mass/time) by the difference between the ambient water quality standard for that pollutant (the maximum acceptable concentration C_max_, mg l^-1^) and its natural concentration in the receiving water body (C_nat_, mg l^-1^) [26].

$${AWU}_{grey}=\frac{L_{leached}}{(C_{max}-C_{nat})\times 10^{-6}}$$ (10)

The accumulated salts/pollutants generally consist of fertilizers, pesticides, and insecticides. In this study, the Nitrate (N) was the representative pollutant, and α was valued at 10% flat rate based on the study of Hoekstra and Chapagain [8]. Subsequently, C_nat_ was considered as being equal to zero and C_max_ as being equal to 11.5 mg l^-1^ as per the Saudi water quality standards [27]. The magnitude of N leaching, however, depends on soil conditions (irrigation frequency, rainfall pattern, soil texture, percolation rate, etc.) and methods of fertilizer application, including rate, time and agronomical practices [37]. Based on the chemical application rate per hectare (AR, kg ha^-1^) and the leaching fraction (α), the L_leached_ can be estimated using Eq (11) according to Chapagain and Hoekstra [37].

$$L_{leached}=\alpha \times L_{application}$$ (11)

#### Assessment of water footprint (WF_Agro_)

Water Footprint (WF) of both maize and carrot crops was calculated, based on the framework explained in Chapagain and Hoekstra [37], as the ratio of the total water used (m^3^ ha^-1^) and the crop yield (t ha^-1^). The three components, WF_Agro-b_ originated from irrigated water, WF_Agro-g_ originated from rainwater and, the WF_Agro-gr_ related to leaching of salts and wash out agro-chemicals [8,19] estimated and subsequently, the total water footprint (WF_Agro_) of silage maize and carrot crops was calculated as in Eq (12). The obtained WF of silage maize and carrot crops was expressed in m^3^ t^-1^.

$${WF}_{total}={WF}_{g}+{WF}_{b}+{WF}_{gr}$$ (12)

The WF_g_, which is the WF components produced from the green portion (i.e. rainfall) of crop water use, was calculated by dividing the component in crop water use (CWU_green_, m^3^ ha^-1^) by the crop yield (Y, t ha^-1^) as in Eq (13). The WF_b_ and the WF_g_ were calculated in a similar way as in Eqs (14) and (15).

$${WF}_{g}=\frac{{CWU}_{green}}{Y}$$ (13)

$${WF}_{b}=\frac{{CWU}_{blue}}{Y}$$ (14)

$${WF}_{gr}=\frac{{AWU}_{grey}}{Y}$$ (15)

where, WF_b_ and WF_g_ are the WF components produced from the portion of the irrigated water and the chemicals assimilated water, respectively.

### Remote sensing approach

A total of 32 cloud-free Landsat-8 satellite images for the period from December 2015 to December 2016 were downloaded from the portal of the USGS Earth Explorer (http://earthexplorer.usgs.gov). The acquired images (Path 164 and 165; Row 45) covered the entire growth period of carrot and silage maize crops. In order to generate a reliable WF of carrot and silage maize crops, Landsat-8 data were analyzed for soil-adjusted vegetation index (SAVI), land surface temperature, a fraction of ET (ET_f_) and actual ET (ET_a_). Initially, Landsat-8 data were geo-referenced to the Universal Transverse Mercator (UTM) map projection with the World Geodetic System 84 (WGS84) datum. Subsequently, Landsat-8 digital numbers were converted to spectral radiance and transformed to Top-Of-Atmosphere (TOA) reflectance [38,39]. The spectral reflectance and land surface temperature products were generated by executing the “ATCOR” module of Geomatica software program (ver. 2015).

#### Landsat-8 derived evapotranspiration (ET_a_)

Simplified surface energy balance (SSEB) model was used for estimating ET_a_ (Eq 16) of carrot and silage maize in the study area. Pre-defined hot and cold conditions of each pixel were used, in Eq 15, to compute the ET_f_ as described in Senay et al. [40].

$${ET}_{a}={ET}_{f}\times k\times {ET}_{o}$$ (16)

where ET_o_ is the reference ET (mm d^-1^), ET_f_ is the ET fraction (unitless), and *k* is a scaling coefficient (unitless). The ET_o_ was obtained from the weather datasets of the Eddy covariance (EC) system. In this study, *k* value was set to 1 as described in Senay et al. [40] and ET fraction (ET_f_) was computed as per Eq 17.

$${ET}_{f}=\frac{T_{h}-T_{s}}{T_{h}-T_{c}}$$ (17)

where, *T*_*h*_ is the reference hot pixel temperature (K), *T*_*s*_ is the land surface temperature (K) obtained from Landsat-8 images, and *T*_*c*_ is the reference cold pixel temperature (K).

Satellite image analysis was carried out with the help of Geomatica software program (ver. 2015). Each individual Landsat-8 scene was processed separately for computing ET_a_ on the day of satellite overpass. All the available daily ET_a_ images were used for upscaling to the entire season level in order to compute ET_a_ with respect to the length of the growth period (*lgp*) of each crop (Eq 18).

$${ET}_{lgp}=\sum_{i=1}^{n}\left({ET}_{oi}\times {ET}_{fi}\right)$$ (18)

where, *ET*_*lgp*_ is the crop specific *lgp* total ET (mm d^-1^), *ET*_*oi*_ is the reference ET (mm) for period *i* (days) and *ET*_*fi*_ is the representative *ET*_*f*_ (unitless) for the period *i*.

The mean monthly ET, derived from Landsat-8 products, was used in the estimation of the WF of carrot and silage maize crops. Landsat-8 estimated water use (ET_a_) and crop yield were compared with the actual values (WF_Agro_), which were obtained through the agrometeorological method.

#### Landsat-8 based water footprint (WF_RS_)

After computing the *ET*_*lgp*_ of each crop, based on the precipitation days and irrigation, the ET_lgp_ was attempted to segregate into blue and green components of ET_a_. Due to the fact that the study area was low in its annual precipitation (~90 mm y^-1^), the green component of ET_a_ was at its minimum values. The availability of satellite images on rainy days is limited. Hence, the Landsat-8 based WF estimation was limited to only the blue component of ET_a_ (i.e. WF_RS-b_). Subsequently, the WF_RS-b_ was computed as the ratio between the crop water use (i.e. Landsat-8 estimated ET_a_ of blue portion = CWU_blue_) and the predicted yield (Y_P_) as in Eq 14.

### Accuracy assessment

The accuracy of Landsat-8 derived WF_RS-b_ was assessed against the agrometeorological computed WF_Agro-b_. The performance indicators that were used for the accuracy assessment included Pearson correlation coefficient (R^2^), root means square error (RMSE), mean bias error (MBE) and Nash-Sutcliff Efficiency (NSE).

## Results and discussion

### Meteorological conditions

The average monthly meteorological data for the period from December 2015 to December 2016 are presented in Table 2. On the average, the monthly minimum, maximum and mean annual air temperatures were 19.3 °C, 34.0 °C and 26.5 °C, respectively. The total amount of rainfall during the study period was recorded at 13.6 mm. In addition, the average monthly wind speed was 5.0 m S^-1^; however, the average monthly ET_o_ values during the study period ranged between 155 mm (November 2016) and 530 mm (July 2016), with an average daily ET_o_ of 11.0 mm.

**Table 2:** Details of the meteorological parameters during the study period (December 2015 to December 2016).

Year	Month	Temperature (°C)			Rainfall (mm)	Wind Speed (m s^-1^)	ET_o_ (mm month^-1^)	ET_o_ (mm d^-1^)
		Min.	Max	Average				
2015	December	12.2	22.8	17.6		2.8	192	6.9
2016	January	11.6	22.9	17.3		3.6	170	5.7
	February	13.0	25.3	19.0		4.2	234	7.8
	March	18.8	30.7	24.8		3.8	341	11.4
	April	21.1	33.9	27.5	8.9	7.9	336	11.2
	May	27.9	42.1	35.0		6.8	483	16.1
	June	29.2	45.6	37.4		7.2	496	16.5
	July	28.2	46.0	37.1		6.7	530	17.7
	August	25.6	44.6	35.2		5.9	506	16.9
	September	22.9	41.9	32.4		4.8	392	13.1
	October	16.1	34.9	25.2		3.2	246	8.2
	November	12.5	27.1	18.7	4.7	4.6	155	5.2
	December	12.0	24.6	17.1		3.8	189	6.3
Average		19.3	34.0	26.5	13.6	5.0	328.5	11

### Electrical conductivity (EC) for soil and irrigation water

Electrical conductivity (EC) can provide accurate estimates of the number of salts presented in soil and water. The EC of both the soil and irrigation water correlates significantly with other agricultural field properties that affect crop productivity such as soil texture, cation exchange capacity (CEC), drainage conditions, organic matter level. To maintain the quality of soil, it is necessary to leach out salts from the root zone by means of additional irrigation water. For the determination of the Leaching factor (α) as in Eq (4), the soil EC (EC_e_) and irrigation water EC (EC_w_) were used as inputs (Table 3). The values of EC_e_ for carrot fields (3-5-S and 5-5-S) were determined at 1.43 (±0.33) dS m^-1^ and 2.76 (±1.11) dS m^-1^, respectively. On the other hand, the EC_e_ of silage maize fields varied from 2.27±0.48 dS m^-1^ (PAL field) to 5.21±1.26 dS m^-1^ (TE-2 field). The mean EC_w_ of irrigation water in the experimental fields ranged between 1.48 dS m^-1^ (3-5-S field) and 2.09 dS m^-1^ (TE11 field).

**Table 3:** Experimental field soil and irrigation water electrical conductivity.

Field ID	EC_e_				EC_w_		Field ID	EC_e_				EC_w_	
	8 cm depth		1 m depth					8 cm depth		20 cm depth			
	Mean	SD	Mean	SD	Mean	SD		Mean	SD	Mean	SD	Mean	SD
**TE-2**	5.21	1.26	5.16	1.41	1.81	0.30	**P 3–5 (N)**	1.77	0.21	2.61	0.74	1.63	0.21
**TE-9**	2.34	0.33	2.38	0.41	2.04	0.33	**P 3–5 (S)**	1.43	0.33	2.39	0.67	1.48	0.33
**TE-11**	4.27	1.49	3.86	0.61	2.09	0.08	**P-5-5 (N)**	1.76	0.73	2.28	0.67	1.56	0.26
**PAL**	2.27	0.48	2.44	0.28	1.86	0.09	**P-5-5 (S)**	2.76	1.11	2.54	0.73	1.71	0.04

### Crop water requirements

Since groundwater is the main source of irrigation, crops are cultivated throughout the year depending on the demand and price. As part of agrometeorological estimation of WF_Agro_, the computation of crop water requirement (CWR) is essential for accurate scheduling of irrigation water. For the application of irrigation water through the sprinkler system, the irrigation interval and the amount of water applied to the experimental crops (carrot and silage maize) were calculated using CROPWAT software program (Ver. 8.0). As illustrated in Table 4, the CWR varies across the crops and lengths of growth period (*lgp*). The temporal dynamics of ET_o_ and salinity of soil and water plays a key role in the variability of CWR. The CWR together with Leaching requirement (LR) for the summer grown silage maize crop, was estimated as high (1622 mm) as compared to spring grown the crop (1359 mm). Similarly, the CWR+LR of carrot crop grown in summer months of 3243 mm was higher than that of the winter-grown carrot of 620 mm. The agro-climatic variables, such as wind speed and temperature, significantly influenced the ET_o_ and the *lgp* of a crop and the respective K_c_ values, which were the main cause of the increase in CWR of crops in summer.

**Table 4:** Season-wise crop water requirement (CWR) of carrot and silage maize.

Crop	Field ID	Season	Sowing	Harvesting	*lgp* (days)	LR (mm)	CWR mm	CWR+LR mm
Silage Maize	TE-11	Spring	7-Apr-16	27-Jun-16	80	159	1304	1463
	PAL	Spring	10-Apr-16	29-Jun-16	80	318	1304	1622
	TE-2	Summer	26-Jul-16	25-Oct-16	90	92	1140	1232
	TE-9	Summer	26-Jul-16	25-Oct-16	90	287	1072	1359
Carrot	3–5 (N)	Summer	2-May-16	8-Sep-16	130	732	2511	3243
	3–5 (S)	Winter	3-Oct-16	24-Dec-16	90	307	620	927
	5–5 (N)	Summer	22-Jun-16	30-Oct-16	140	620	2169	2788
	5–5 (S)	Winter	24-Dec-15	18-Mar-16	85	135	823	958

### Crop yield

**Fig 3:**
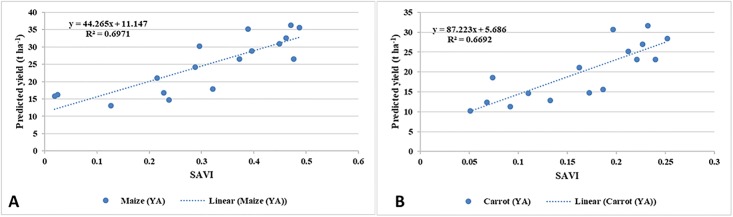
Actual versus predicted yield for the two crops.

Silage maize and carrot yields predictive regression models were developed based on the values of the Soil Adjusted Vegetation Index (SAVI). The SAVI was computed for each crop throughout its growth period from seedling to seven days prior to the harvest. Season and crop wise datasets, about 6 to 7 Landsat-8 images of multiple dates were analyzed for this purpose (S1 File). Subsequently, the obtained SAVI is correlated with the field-recorded yields (Y_A_) for the prediction of yield (Y_P_). A linear relationship between the Landsat-8 derived SAVI and Y_A_ of tested crops were developed, and the best-fit model was used in the prediction of Y_P_ (Fig 3).

A total of 100 samples, i.e. 40 (from two carrot fields) and 60 (from two silage maize fields) sampling locations were identified for each season and the Y_A_ measurements was recorded. Of which, about 60 points (60%) used for the development of the Y_P_ models, while the remaining 40% (40 samples) were used for the cross-validation of the models. The SPSS statistical software (Ver. 18.1) was used for the development and cross-validation of the Y_P_ models.

As shown in Table 5, the obtained models were validated for their accuracy against the in-situ yields using performance indicators, such as Pearson correlation coefficient (R^2^), root mean square error (RMSE), mean bias error (MBE) and Nash-Sutcliff Efficiency (NSE). The best relationship between crop yield and SAVI was obtained when the crops were in mid-stage of development. In the case of silage maize, the best response was observed on the Julian days of 162 (SAVI = 0.456) and 272 (SAVI = 0.462) for summer and spring seasons, respectively, when the crops were at their peak growth stage. The best response for carrot fields was observed on the Julian days of 68 (winter) and 146 (summer), when the values of SAVI were estimated at 0.416 and 0.398, respectively. The average predicted yield (Y_P_, DM t ha^-1^), for silage maize and carrot, was 31.98 and 37.65, respectively. The variation between Y_A_ and Y_P_ was found to be 15% (i.e. 4.5 t ha^-1^) for carrots and 17% (5.4 t ha^-1^) for silage corn.

**Table 5:** The accuracy of the developed crop yield models.

Crop	Model (Y = crop yield, t ha^-1^)	Model Validation				Cross Validation			
		R^2^	RMSE (%)	NSE	MBE (%)	R^2^	RMSE (%)	NSE	MBE (%)
**Silage Maize**	Y = 44.265 × SAVI + 11.147	0.70	9.6	0.62	-6.2	0.74	10.8	0.62	2.4
**Carrot**	Y = 87.223 × SAVI + 5.686	0.67	10.2	0.69	2.9	0.72	9.4	0.46	-1.6

### Crop water use (CWU)

The CWU of carrot and silage maize crops was obviously observed to vary across the seasons. The pattern of the CWU (green, blue and grey) and the leaching fraction (pollutant load for the dilution of the applied nutrient salts) across the experimental fields were presented, along with the actually applied irrigation, in Table 6.

**Table 6:** Field and season wise actual applied water, crop water use (CWU), leaching fraction and Landsat-8 predicted CWU_blue_.

Crop	Pivot	Applied Water (mm)	L_leached_ (mm)	CWU (mm)				RS based CWU (ET, mm)
				Green	Blue	Grey	Total	
Silage Maize	TE-11	1232	0.16	9	830	210	1049	725
	Palace	1688	0.28	0	1053	352	1405	1212
	TE-2	1072	0.11	0	837	109	946	1072
	TE-9	1098	0.3	0	676	320	996	685
Carrot	3–5 (N)	1775	0.2	0	1037	420	1457	1172
	3–5 (S)	836	0.47	5	374	360	739	768
	5–5 (N)	2684	0.32	0	1473	680	2153	1341
	5–5 (S)	1048	0.32	0	638	182	820	865

**Fig 4:**
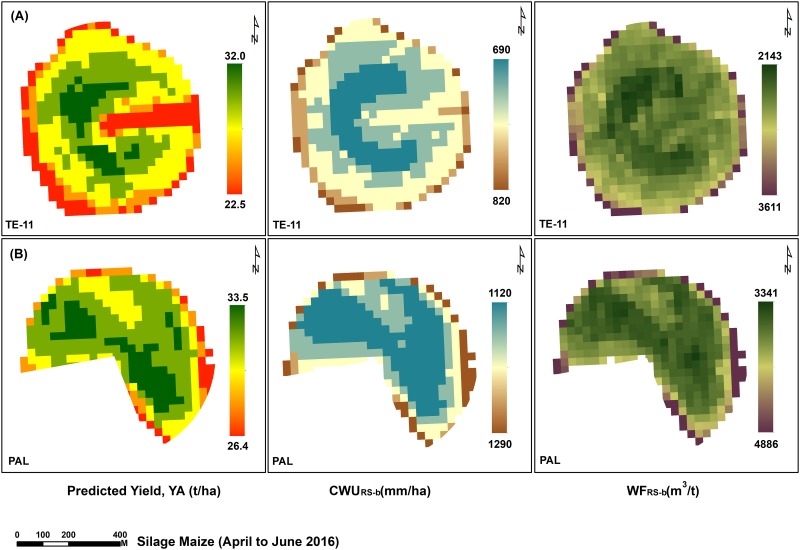
Landsat-8 derived yield, CWU and WF of silage maize cultivated in TE-11 and PAL fields.

**Fig 5:**
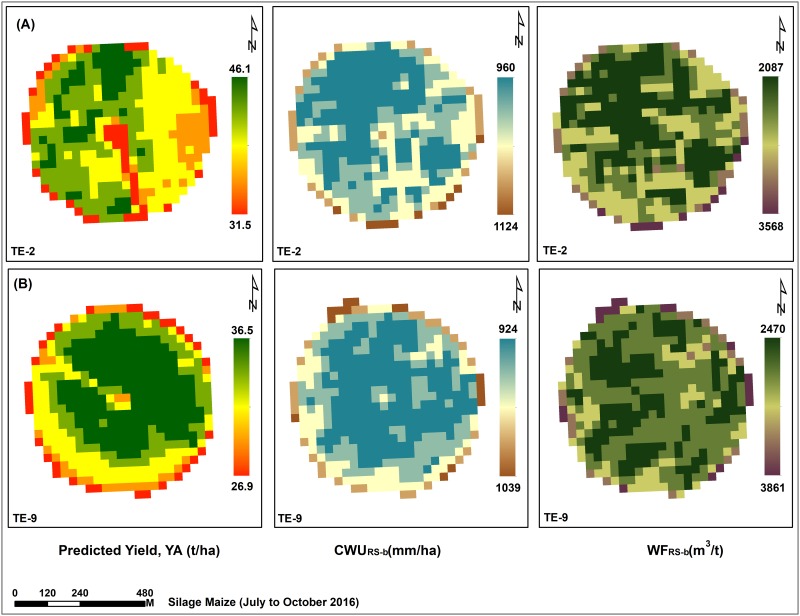
Landsat-8 derived yield, CWU and WF of silage maize cultivated in TE-2 and TE-9 fields.

**Fig 6:**
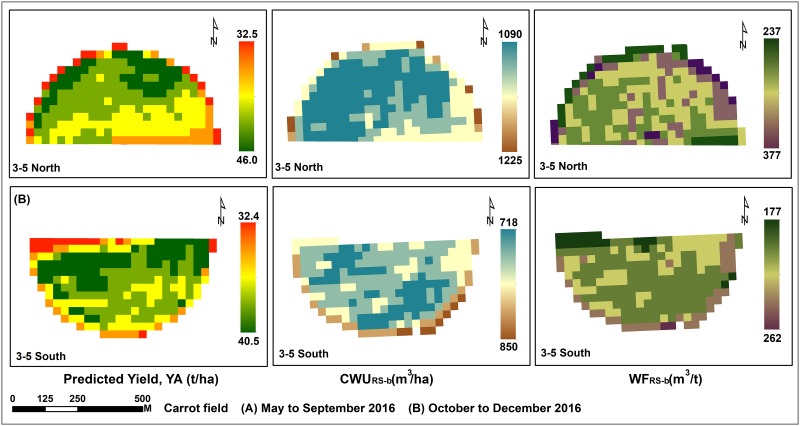
Landsat-8 derived yield, CWU and WF of carrot crop cultivated in the field number 3–5.

**Fig 7:**
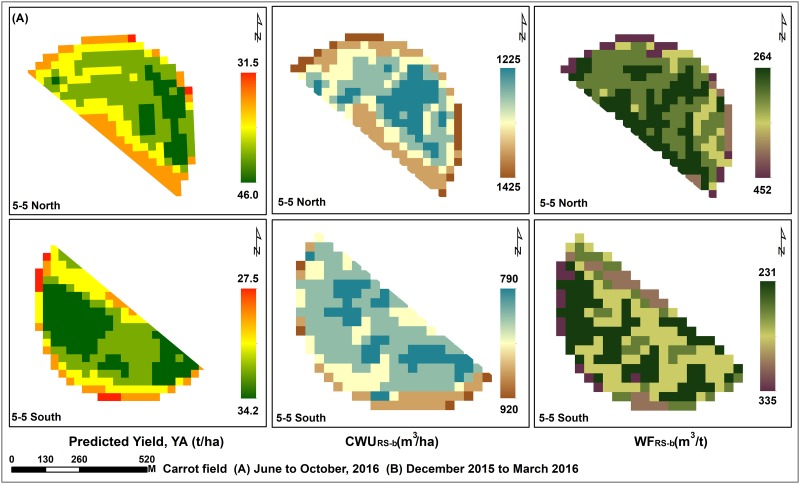
Landsat-8 derived yield, CWU and WF of carrot crop cultivated in the field number 5–5.

Although the study farm was keen to achieve the best yields through effective management of irrigation water, there was a reduction ranging between 5.12% and 39.8% in actually applied irrigation water compared to the estimated CWR for the two experimental crops (Table 4). The highest reduction was observed during the summer season, where 39.8% and 19% reduction occurred for silage maize and carrot, respectively. The green, blue and grey components of CWU (mean) for carrot fields were 0.4%, 68.1% and 31.8%, respectively. For silage maize crop, these values were 0.81%, 77.25% and 22.54%. In order to understand the spatial variation in WF, Landsat-8-based CWU_RS-b_ (ET) of the two crops was predicted (Figs 4 to 7). The predicted CWU_RS-b_ for silage maize was ranged from 712 (winter) up to 1108 (summer), while for carrot crop it was about 543 and 1382 mm for both summer and winter seasons, respectively. The RMSE (mm) between CWU_Agro-b_ and CWU_RS-b_ was ranged from 75.49 (carrot) to 234.37 (maize).

### Water footprint (WF)

The patterns of WF (green, blue and grey) are presented in Table 7. Among the four silage maize investigated fields, filed number TE-11 was the lowest in WF_Agro_ (3545 m^3^ t^-1^); while the highest WF_Agro_ (4960 m^3^ t^-1^) was recorded for field number PAL. On the average, the contribution of WF_Agro-g_, WF_Agro-b_ and WF_Agro-gr_ for silage maize was estimated at 0.75%, 77.33% and 22.48%, respectively. The WF_Agro_ of carrot crop ranged was ranged between 297 m^3^ t^-1^ (field number 3-5N) and 502 m^3^ t^-1^ (field number 5-5N), with an average WF_Agro_ value of 396 m^3^ t^-1^ over the entire study period. The WF_Agro-g_, WF_Agro-b_ and WF_Agro-gr_ of carrot were contributed with 0.6%, 66.9% and 32.9%, respectively. The remote sensing (RS) based WF_RS-b_ varied across the crops from 276 (±73) m^3^ kg^-1^ (carrot) to 2884 (±441) m^3^ t^-1^ (silage maize). The RS based yield (Y_P_), WF_Agro_ and WF_RS-b_ are provided in Table 7. The variation between WF_RS-b_ and WF_Agro-b_ was about 17% and 14% for silage maize and carrot, respectively.

**Table 7:** The water footprint (WF) of silage maize and carrot crops.

Crop	Pivot	Y_A_ (t ha^-1^)	Water Footprint (m^3^ t^-1^)				Y_P_ (t ha^-1^)	WF_RS-b_ (m^3^ t^-1^)
			WF_Agro-g_	WF_Agro-b_	WF_Agro-gr_	WF_Agro_		
Silage Maize	TE-11	29.59	30.08	2805	710	3545	28.70	2526
	PAL	28.32	0	3717	1243	4960	31.71	3822
	TE-2	25.37	0	3300	430	3730	40.68	2635
	TE-9	26.07	0	2593	1227	3820	26.83	2553
	Mean	27.34	30.08 (0.75%)	3104 (77.33%)	902 (22.48%)	4014	31.98	2884
Carrot	3–5 (N)	40.11	0	211	86	297	45.60	257
	3–5 (S)	26.95	2.48	197	190	390	31.68	242
	5–5 (N)	42.85	0	344	159	503	44.69	300
	5–5 (S)	20.68	0	309	88	397	28.63	302
	Mean	32.64	2.48 (0.60%)	265 (66.9%)	131 (32.9%)	396	37.65	276

The WF_Agro_ composed with the highest fraction of the WF_Agro-b_ from 67% for carrot and 77% for maize crop. The grey water, however, corresponded to local salinity and crop salt tolerance and ranged from 22% (maize) to 33% (carrot). The current results for carrot crop indicated that the obtained WF was 30% lower than that reported by Multch et al [26]. Results of silage maize, however, indicated that the obtained WF was 2.5 times lower than that reported by Chowdhury et al. [36]. The total WF_Agro_ values of silage maize (4014 m^3^ t^-1^) and carrots (396 m^3^ t^-1^) obtained in this study were lower than that of earlier studies (Table 8). However, the WF_Agro-gr_ determined in this study was observed to be higher than the global averages stated in Mekonnen and Hoekstra [16] for both silage maize and carrot crops. Results of this study indicated that WF_Agro_ was relatively higher than the global WF statistics [16]. This may be due to the fact that global statistics utilized both irrigated and arid crops for the compilation of global WF.

**Table 8:** Obtained and reported WF values.

Study	Silage Maize WF (m^3^ t^-1^)				Carrot WF (m^3^ t^-1^)			
	Green	Blue	Grey	Total	Green	Blue	Grey	Total
Present Study (WF_Agro_)	30	3104	902	4014	2	265	131	396
Multch et al. [26]	154	3566	1041	4751	23	427	67	517
Mekonnen and Hoekstra [16]	947	249	212	1222	106	28	61	195

**Fig 8:**
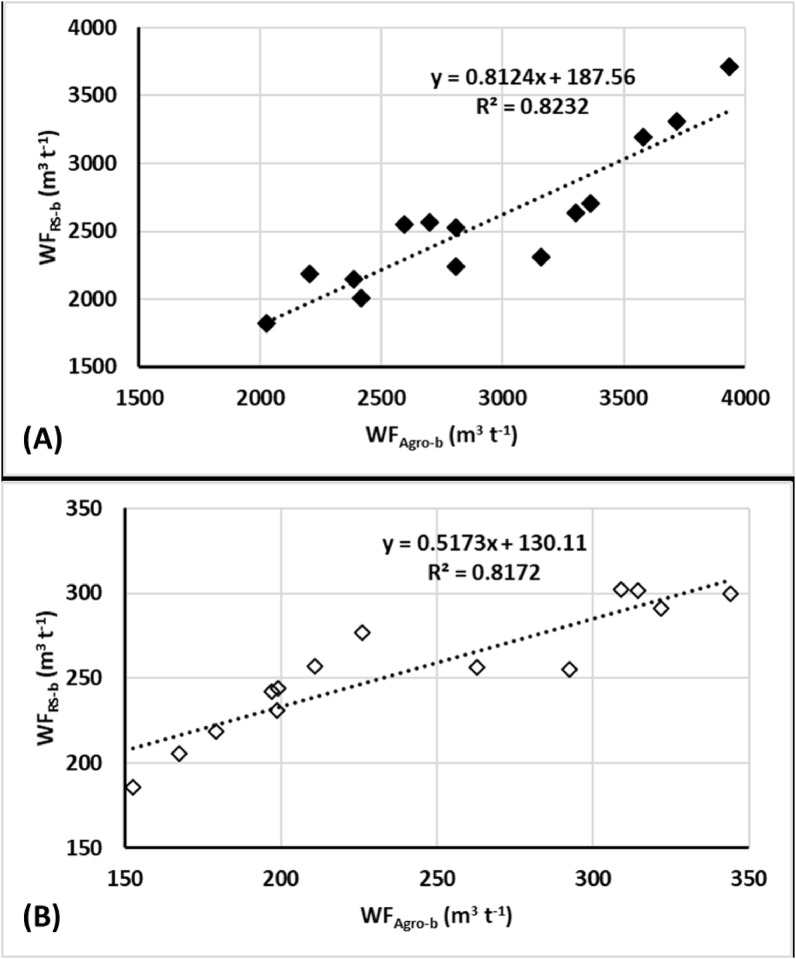
Remote sensing versus empirical approach based estimated blue component of WF (WF_Blue_): (A) silage corn and (B) carrot crops.

The empirical (agro-meteorological) approach based WF_Agro-b_, calculated from actual in-situ data, was used as a reference against which the accuracy of the Landsat-8 determined WF_RS-b_ was evaluated. A comparative analysis of the blue component of WF (WF_b_) estimated by both WF_Agro-b_ and WF_RS-b_ approach is illustrated in Fig 8. Results revealed a highly significant linear relationship between the empirical (WF_Argo-b_) and the Landsat-8 derived WF_RS-b_. In the case of silage maize, the recorded R^2^ was 0.82 (P>F = 0.010) with the RMSE value of 501 m^3^ t^-1^ (17%) and an MBE value of 218 m^3^ t^-1^ (8%). For the carrot crop, the R^2^ found to be 0.82 (P>F = 0.049) the RMSE and MBE values were 39.21 m^3^ t^-1^ (14%) and 10.39 m^3^ t^-1^ (4%), respectively. The NSE for silage corn and carrot crops was 0.98 and 0.96, respectively. Senay et al. [40] found that the SSEB model was able to capture the seasonal ET_a_ well with a strong correlation, where the R^2^ value ranged between 0.76 (carrot) and 0.97 (silage maize) for the relationship between model estimated and actual ET. However, in terms of magnitude, the specific agreement in overestimation or underestimation of ET values may depend on the seasonal dynamics of climate and vegetation cover. Particularly, the extreme ET is usually reliable since the subjective selection of hot and cold reference pixels is eliminated and the only variable from season to season is the land surface temperature.

On the other hand, irrigation efficiency at the border areas of the center pivots depends mainly on the system efficiency along with the soil type, slope, and surface roughness. In this study, the boarder-effect is minimal as the fields are having slopes of less than 5%. However, the deposition of fine sand as a result of sand dunes and the prevailing winds from the surroundings of the “desert area” resulted in the variation of the WF at the border areas.

## Conclusions

A field study was conducted to investigate the Water Footprint (WF) for carrot and silage maize crops cultivated in Saudi Arabia during the period from December 2015 to December 2016. The specific conclusions drawn from the study are as follows:

- This study demonstrated the methods of estimating water footprint using Eddy covariance (WF_Agro_) and remote sensing-based approach for WF mapping from Landsat-8 multispectral imagery.
- Due to the limited availability of satellite images on rainy days, the Landsat-8 based estimates were restricted to only the blue component of WF (i.e. WF_RS-b_).
- The utility of Landsat-8 data in mapping CWU_RS-b_ showed reliable seasonal estimates of 1199 mm (summer) and 761 mm (spring/winter), which were in accordance with the Eddy covariance measured ET at 939 mm and 750 mm for summer and spring/winter growth periods, respectively.
- Among the six experimental fields, Landsat-8 determined WF_RS-b_ of silage maize varied from 2526 to 3822 m^3^ t^-1^. For carrot, WF_RS-b_ values were estimated at 242 to 302 m^3^ t^-1^.
- The Landsat-8 derived WF_RS-b_ showed a highly significant linear relationship with the empirical WF_Agro-b_ approach (R^2^ = 0.77, P>F = 0.001).
- Feasible water footprint assessment system of agricultural crops for the efficient use of available water resources was developed by the integration of remote sensing technology (Landsat-8 satellite images) and weather data from the agro-meteorological station (Eddy Covariance system).

## Supporting information

S1 File Crop water requirement, Landsat-8 data and SAVI. (PDF)
