# Correction: Utilization of Landsat-8 data for the estimation of carrot and maize crop water footprint under the arid climate of Saudi Arabia

**DOI:** 10.1371/journal.pone.0198378

**Published:** 2018-05-24

**Authors:** The PLOS ONE Staff

There is an error in affiliation 1 for all authors. Affiliation 1 should be:

Precision Agriculture Research Chair, King Saud University, Riyadh, Saudi Arabia.

The publisher apologizes for the error.
