# Supplementary material for: Utilization of Landsat-8 data for the estimation of carrot and maize crop water footprint under the arid climate of Saudi Arabia
Source: PLoS One. 2018 Feb 12;13(2):e0192830. doi: 10.1371/journal.pone.0192830 (PMC5809052; doi:10.1371/journal.pone.0192830)
Supplement: S1 File — (PDF) [file pone.0192830.s001.pdf]

Supplementary data (S1 File)

Calculation of Crop Water Requirement (mm) based on the historical data recorded between 1990 and 2015 - Extract from CROPWAT software

Field ID	CROP	Leaching Factor	Leaching Requirement	2015	2016												CWR (MM)	CWR + LR (MM)
				DEC.	JAN.	FEB.	MAR.	APR.	MAY	JUNE	JULY	AUG.	SEP.	OCT.	NOV.	DEC.		
TE11	Maize	0.11	159				22.1	64.5	542.8	674.7							1304	1463
PAL		0.20	318				22.1	64.5	542.8	674.7							1304	1622
TE-2		0.07	92								18	388	604	130			1140	1232
TE-9		0.21	287								8	388	604	72			1072	1359
3-5 (N)	Carrot	0.23	732						257	531	772	766	185				2511	3243
3-5 (S)		0.33	307											128	312	180	620	927
5-5 (N)		0.22	620							81.3	363.2	694.5	648.9	380.9			2169	2788
5-5 (S)		0.14	135	32.6	184.1	330.1	276.4										823	958

Actual water discharged through the Centre pivot - Irrigation pattern prepared based on the ET and Weather parameters recorded during the study period

Field ID	CROP	Area (ha)	GPM	2015	2016												TOTAL (T/HRS.)	WATER APPLIED (MM)
				DEC.	JAN.	FEB.	MAR.	APR.	MAY	JUNE	JULY	AUG.	SEP.	OCT.	NOV.	DEC.		
				T/HRS.	T/HRS.	T/HRS.	T/HRS.	T/HRS.	T/HRS.	T/HRS.	T/HRS.	T/HRS.	T/HRS.	T/HRS.	T/HRS.	T/HRS.		
TE11	Maize	50	1250				396	560	728	472							2156	1231.62
PAL		37	1450				312	486	711	376							1885	1687.97
TE-2		50	1250								24	730	700	422			1876	1071.67
TE-9		50	1300								24	730	696	399			1849	1098.49
3-5 (N)	Carrot	20	500						730	730	730	730	188				3108	1775.45
3-5 (S)		20	500											726	408	329	1463	835.74
5-5 (N)		20	700							516	727	727	684	702			3356	2683.96
5-5 (S)		20	700	259	352	335.5	364.5										1311	1048.47

Variation in CWR + LR and actual applied irrigation water

Field ID	CROP	Required Irrigation (MM)			WATER APPLIED (MM)	Variation in water application		Inference
		LR (MM)	CWR (MM)	LR + CWR		MM	%	
TE11	Maize	159	1304	1463	1232	281	19	
PAL	Maize	318	1304	1622	1688	-152	-10	
TE-2	Maize	92	1140	1232	1072	244	19	
TE-9	Maize	287	1072	1359	1098	168	13	
3-5 (N)	Carrot	732	2511	3243	1775	1199	40	Exceeded the capacity of irrigation system
3-5 (S)	Carrot	307	620	927	836	-83	-11	
5-5 (N)	Carrot	620	2169	2788	2684	-117	-5	
5-5 (S)	Carrot	135	823	958	1048	-88	-9	

Calculation of Crop Water Use (mm) using the Eddy covariance and weather station recorded datasets (Dec. 2015 to Dec. 2016) - AGROMETEOROLOGICAL APPROACH (CWU_{Agro})

Field ID	CROP	CWU_{Agro-g}	$CWU_{Agro-gr}$	CWU_{Agro-b}														CWU_{Agro} (mm)
				2015	2016													
				DEC	JAN	FEB	MAR	APR	MAY	JUNE	JULY	AUG	SEP	OCT	NOV	DEC	Total	
TE11	Maize	8.90	210				7	282	362	179							**830**	**1049**
PAL			352				5	269	435	344							**1053**	**1405**
TE-2			109								34	304	361	139			**837**	**946**
TE-9			320								21	278	298	79			**676**	**996**
3-5 (N)	Carrot		420						140	261	283	301	52				**1037**	**1457**
3-5 (S)		4.70	360											133	124	117	**374**	**739**
5-5 (N)			680							89	424	455	314	190			**1473**	**2153**
5-5 (S)			182	40	167	234	198										**638**	**820**

Details of Landsat-8 Images (Date of Pass, path and row) used in the study												
Landsat-8 data (Path: 164 & 165 Row: 43 & 44)												
2015	2016											
26-Dec-15	3-Jan-16	4-Feb-16	23-Mar-16	8-Apr-16	3-May-16	4-Jun-16	6-Jul-16	7-Aug-16	8-Sep-16	1-Oct-16	18-Nov-16	13-Dec-16
	12-Jan-16	13-Feb-16		17-Apr-16	19-May-16	11-Jun-16	13-Jul-16	14-Aug-16	15-Sep-16	10-Oct-16		20-Dec-16
	19-Jan-16			24-Apr-16	26-May-16	20-Jun-16	22-Jul-16	30-Aug-16	24-Sep-16	17-Oct-16		
	28-Jan-16					27-Jun-16	29-Jul-17			26-Oct-16		

Field ID	CROP	Season	SAVI (Landsat-8 data)							
			I1	I2	I3	I4	I5	I6	I7	Cumulative
TE11	Maize	Spring	0.05	0.12	0.16	0.34	0.88	0.53	0.49	2.57
PAL	Maize	Spring	0.05	0.10	0.14	0.30	0.76	0.46	0.43	2.24
TE-2	Maize	Summer	0.07	0.19	0.25	0.42	0.82	0.46	0.38	2.59
TE-9	Maize	Summer	0.06	0.14	0.18	0.35	0.82	0.48	0.43	2.47
3-5 (N)	Carrot	Summer	0.06	0.24	0.31	0.40	0.62	0.72	0.56	2.90
3-5 (S)	Carrot	Winter	0.06	0.26	0.34	0.44	0.67	0.78	0.61	3.17
5-5 (N)	Carrot	Summer	0.07	0.28	0.36	0.46	0.72	0.83	0.65	3.37
5-5 (S)	Carrot	Winter	0.06	0.27	0.35	0.45	0.68	0.78	0.63	3.23
